# Enhanced Pervaporation Properties of PVA-Based Membranes Modified with Polyelectrolytes. Application to IPA Dehydration

**DOI:** 10.3390/polym12010014

**Published:** 2019-12-19

**Authors:** Mariia Dmitrenko, Anna Kuzminova, Andrey Zolotarev, Sergey Ermakov, Denis Roizard, Anastasia Penkova

**Affiliations:** 1 St. Petersburg State University, 7/9 Universitetskaya nab., St. Petersburg 199034, Russia; m.dmitrienko@spbu.ru (M.D.); ai.kuzminova@mail.ru (A.K.); andrey.zolotarev@spbu.ru (A.Z.); s.ermakov@spbu.ru (S.E.); 2 Laboratoire Réactions et Génie des Procédés, CNRS, Université de Lorraine, ENSIC, 1 rue Granville, 54000 Nancy, France; denis.roizard@univ-lorraine.fr

**Keywords:** polyvinyl alcohol, polyelectrolyte, pervaporation, ultrafiltration, layer-by-layer deposition, isopropanol

## Abstract

In this work, dense and supported pervaporation polyvinyl alcohol (PVA)-based membranes modified with poly(allylamine hydrochloride) (PAH) and poly(sodium 4-styrenesulfonate)(PSS)/PAH top nanolayers were synthesized. Two main points were investigated: the role of the polyelectrolyte PAH on water selectivity of the selective polymer matrix and the impact of the porous substrate based on polyacrylonitrile (PAN) and aromatic polysulfone amide (UPM-20^®^), used to get supported high-performance membranes. Various methods of analysis (fourier-transform infrared spectroscopy (FTIR), scanning electron microscopy (SEM), atomic force microscopy (AFM), small-angle X-ray scattering (SAXS), porosity, contact angles, ultrafiltration) were applied to study the developed membranes. Transport characteristics of the developed membranes were studied in isopropanol dehydration by pervaporation. Obtained results are discussed in the light of the structure and physicochemical characteristics of these PVA/PAH membranes and the types of porous substrate. It was shown that the PAN-supported membrane with the selective layer based on PVA/PAH modified by 10 polyelectrolyte PSS/PAH bilayers possessed ~4.5 times higher permeation flux with the same high selectivity level (99.9 wt % water in the permeate) for the dehydration of the isopropanol (20 wt % water) at 60 °C compared to the commercial analog PERVAP^TM^ 1201.

## 1. Introduction

Currently, membrane-based separation technologies are known to be efficient for molecular separations, and they are already considered as alternative processes to traditional methods (absorption, distillation, extractive rectification) at the industrial scale, especially for the separation and purification of azeotropic liquid mixtures and for components having close boiling points. This is because membrane separation technologies offer significant energy savings and can give rise to environmentally friendly processes. Pervaporation is one of the promising classical membrane processes for the dehydration of organic substances, especially alcohols with the use of hydrophilic membranes, because of the high water selectivity of the membranes, equipment compactness, and lower energy consumption [1,2,3,4,5,6,7]. One of the most studied mixtures for dehydration by pervaporation is the isopropanol (i-PrOH)–water system. Isopropanol is an important solvent widely used for production of various chemical reagents and as a substitute for ethanol in areas such as cosmetics, perfumes, household chemicals, disinfectants, repellents, etc. Yet, for industrial application, the solvent must usually be anhydrous. In addition, this alcohol forms an azeotropic mixture with water (12 wt % water–88 wt % isopropanol) [8], which considerably hinders its dehydration via simple traditional methods of separation that entail a non-environmentally friendly process (addition of a third harmful organic reagent) and a high energy consumption (high temperature or low pressure, expensive equipment). Pervaporation is a promising and perspective technology for this task. Different types of pervaporation membranes (polymeric, inorganic, and composite) were developed for the dehydration of isopropanol [9,10,11,12]. Polymeric membranes find wider applications and are attractive because of the simplicity of their fabrication and lower price compared to inorganic membranes. The most popular polymer materials for dehydration are green water-soluble polymers, such as polyvinyl alcohol (PVA), chitosan (CS), cellulose, and sodium alginate [1,13,14], because these polymers possess high selectivity to water. However, the use of membranes based on these polymer materials requires additional cross-linking that, as a rule, leads to the decrease of permeation flux [12]. To improve the transport characteristics, additional bulk and surface modification methods can to be successfully applied, as previously shown [15,16,17,18,19,20].

In this study, the selected material is polyvinyl alcohol, which is widely used for dehydration purposes due to its high water selectivity, good film-forming properties, and economic accessibility [1,4,21,22]. However, pristine PVA membranes without additional modification and cross-linking exhibit poor stability in aqueous solutions where large swelling is observed. This phenomenon makes it impossible to use them for the separation of dilute solutes. 

Earlier studies and literature reviews demonstrated that the application of polyelectrolytes for bulk and surface modification could lead to significant improvement of transport characteristics due to extrinsic and intrinsic charge overcompensation mechanisms and ion pairing, which led to significant changes in free volume and surface hydrophilicity [23,24]. 

One of the modern and versatile methods of membrane surface modification is the layer-by-layer (LbL) assembly technique for the fabrication of ultra-thin defect-free polyelectrolyte (PEL) layers on the surface of the polymer membrane [25]. This method consists of sequential alternating adsorption of polycations and polyanions on a membrane surface and allows one to control the thickness and the surface properties (for example, hydrophilicity, etc.) of membranes. Varying modification conditions such as the number of PEL layers, types of PEL, their ionic strength, and pH of PEL solutions permits flexibly improving the pervaporation characteristics of membranes [26,27,28]. The LbL assembly technique was firstly explored by Hong and Decher in 1991 and reported by Iler in 1996 [25]. Surface modification by LbL deposition of PEL can be carried out via various methods such as spray-LbL, spin-LbL, and dip-LbL. It is one of the common simple ways for modifying polymer membranes due to the formation of denser, thicker, and smoother PEL multilayers [25]. The LbL modification by PEL of PVA polymer is applied in different fields of science and technology [29,30,31]. In Reference [29], a novel high-flux membrane for forward osmosis (FO) was prepared via LbL deposition of three PEL bilayers (chitosan (CS)/polyacrylic acid (PAAc)) on the developed membrane substrate based on a nanocomposite of polyvinyl alcohol (PVA) and montmorillonite clay. The developed FO membranes were tested in deionized water (DI) and synthetic wastewater. It was shown that the application of LbL surface modification of the prepared substrate allowed the achievement of low salt leakage. LbL coating of different bilayer numbers (5, 10, 20) of poly (diallyldimethylammonium chloride)/polystyrene sulfonic acid sodium salt) was also applied for the development of stable anion exchange KOH-doped PVA membranes cross-linked with glutaraldehyde (GA) or poly(ethylene glycol) diglycidyl ether (PEDGE) [30]. It was determined that the ionic conductivity of the membranes increased with the increase in the number of bilayers, and the multilayered film was stable against fuel cell operating conditions in terms of pH. Thus, the application of the LbL technique for anion exchange membranes makes it possible to improve their characteristics and become promising membranes for fuel cell applications. In Reference [31], LbL deposition using 2-anthraquinone sulfonate (2-AQS), 2,6-AQS, and 2,7-AQS with polyethyleneimine (PEI) was used for modification of the surface of poly(vinylalcohol-*co*-ethylene) (PVA-*co*-PE) nanofiber composite membranes to develop membranes with photoinduced self-cleaning functions. The deposition of only one layer of 2,6-AQS was able to provide the expected photoinduced self-cleaning properties, while the increase in the number of bilayers led to an adverse effect on the decomposition efficiency. 

One of the most commonly used cationic polyelectrolytes is poly(allylamine hydrochloride) (PAH) due to its high affinity for water, because of the presence of amine hydrochloride functional groups [32,33]. Polyelectrolyte PAH was already used as a noncovalent agent to functionalize multi-walled carbon nanotubes (MWCNT) for better dispersion into the PVA matrix [34]. Nanocomposite membranes based on PVA/MWCNT–PAH were prepared for pervaporation dehydration of isopropanol, and they were shown to possess improved transport properties compared to the pristine membrane. Namboodiri et al. [32,33] developed new dense membranes based on mixtures of poly(allylamine hydrochloride)–poly(vinyl alcohol) cross-linked by glutaraldehyde (GA) for the pervaporation dehydration of various organic solvents such as ethanol, methanol, acetone, and isopropanol. They incorporated PVA into the PAH matrix at various formulation ratios to get improved membrane characteristics, such as flexibility and stability. 

Our previous studies [23,35] showed that many parameters could be used to tune the PEL effect on the transport characteristics of the PVA membranes, such as the selected pair of polycation–polyanion, the order of their deposition via the LbL deposition technique, the number of cycles, and additional bulk modification (introduction of PAH or CS). Thus, a PVA–fullerenol membrane modified by PAH (4.7 wt %) exhibited the best transport properties for the dehydration of isopropanol after an additional surface modification by 10 bilayers of poly(sodium 4-styrenesulfonate) (PSS)/PAH, which led to an increase in the permeation flux (0.29 kg/(m^2^h)) with a high level of selectivity (water content in the permeate was 98.4 wt %) [23]. PAH was introduced into the PVA matrix in order to improve the dispersion of fullerenol in the membrane matrix, as well as the adhesion of the PEL layers for surface modification. It was also observed that the deposition of PSS/PAH layers onto a non-porous selective PVA layer promoted the stability of PEL layers during pervaporation (nano-sized polyelectrolyte layers were not washed away). On the other hand, the use of porous substrate based on polyethyleneterephthalate with polyacrylonitrile resulted in a limitation of the stability in water of the deposited PSS/PAH nanolayers (up to 20 wt % water in the feed) and the limitation of the number of PEL bilayers (from 15 bilayers) [36]. It should be noted that, in References [23,35], the effect of PAH introduction into the PVA matrix and the interaction nature between PAH and PVA were not provided. Moreover, the selectivity properties of the best PVA–fullerenol (5%)–PAH/LbL-10 membrane could be improved (98.4%). 

The aim of this work was to study dense and supported pervaporation PVA-based membranes modified with poly(allylamine hydrochloride) (PAH) and PSS/PAH top nanolayers. The transport properties of the prepared membranes based on PVA–PAH were studied by pervaporation of an isopropanol–water mixture. Two main points were investigated: (1) the role of the polyelectrolyte PAH on properties of the polymer matrix studied by FTIR spectroscopy, scanning electron microscopy (SEM), atomic force microscopy (AFM), pervaporation, and contact-angle measurements, and (2) the impact of the commercial porous substrates based on polyacrylonitrile (PAN) and aromatic polysulfone amide (UPM-20^®^) investigated by SEM, AFM, the standard porosimetry method, contact-angle measurements, and ultrafiltration for understanding the mass transfer in the pervaporation of isopropanol dehydration. 

In order to assess the promising application of the PAN-supported membranes modified by PEL, its transport properties were studied at different temperatures (20, 30, 45, 60 °C) during pervaporation dehydration of the i-PrOH (20 wt % water), compared with the commercial analog PERVAP^TM^ 1201. Stability of top nanolayers deposited via the LbL technique on the surface of the PAN-supported membrane was studied by contact-angle measurements and SEM.

## 2. Materials and Methods 

### 2.1. Materials

PVA with a molecular weight (*M*_w_) of 141 kDa in the form of powder was obtained from ZAO “LenReaktiv” (certificate of analysis № 553041-3013, date of manufacture September 2011, St. Petersburg, Russia) and used as the membrane material. Maleic acid (MA) purchased from “Sigma-Aldrich” (Nancy, France) with a purity of >99.0% was used as a cross-linking agent for PVA membranes. Poly(allylamine hydrochloride) (PAH, *M*_w_ ~50,000) and poly(sodium 4-styrenesulfonate) (PSS, *M*_w_ ~70,000) as the cationic and anionic polyelectrolytes, respectively, were purchased from “Sigma-Aldrich” (Nancy, France). Isopropanol (i-PrOH) was obtained from “Vekton” (St. Petersburg, Russia), and all the chemicals were used without additional treatment. Hydrophilic commercial porous membranes based on polyacrylonitrile (PAN, Institute “Leibniz-Institut für Polymerforschung Dresden”, Dresden, Germany) and aromatic polysulfone amide (UPM-20, “Vladipor”, Vladimir, Russia) were used as a membrane substrate. The commercial supported membrane “PERVAP^TM^ 1201” (a cross-linked PVA membrane for the dehydration of mixtures containing up to 80 wt % water, Sulzer Chemtec Co., Oberwinterthur, Switzerland) was tested to compare transport parameters. Bovine serum albumin (BSA), purchased from “PanReac AppliChem” (№ A2244,0050: Albumin fraction V (pH 5.2), *M* = 68,000 g/mol, Moscow, Russia), was used as a foulant in the ultrafiltration experiments for the testing of commercial porous substrates.

### 2.2. Membrane Preparation

#### 2.2.1. Dense (Self-Standing) Membranes 

The preparation of the dense membranes based on polymer blend PVA–PAH was carried out according to the following procedure: a 2 wt % aqueous PVA solution was prepared by dissolving in bi-distilled water with constant stirring for 5 h at a temperature of 90 °C. The required amount of poly(allylamine hydrochloride) (PAH) (0 or 4.7 wt % with respect to the weight of the polymer) was dispersed in the filtered prepared PVA solution by ultrasonic treatment for 40 min at ambient temperature. The maximum loading of poly(allylamine hydrochloride) was limited to 4.7 wt % due to its poor dispersion in solutions having higher amounts. All of the membranes were subjected to the chemical treatment according to a previously reported method [37]; maleic acid (MA) (35 wt % with respect to the weight of the polymer) was added to the PVA and polymer blend solutions as an additional cross-linking agent to improve the degree of cross-linking of polymer chains. Chemically cross-linked PVA and PVA–PAH dense membranes were prepared by a solution casting method and evaporation of the solvent in a thermostat at 40 °C for 24 h with further heat treatment at 110 °C for 120 min. The thickness of the membranes was 45 ± 3 µm. 

#### 2.2.2. Supported Membranes

Supported membranes were prepared by casting of 2 wt % aqueous solution of PVA with 35 wt % MA or its polymer blend solution (PVA/PAH (4.7%)/MA (35%)) onto the surface of the porous substrate based on polyacrylonitrile (PAN) or aromatic polysulfone amide (UPM-20) and drying at room temperature for 24 h to evaporate the solvent. A thin fabricated selective layer of these membranes was prepared according to the procedure described for dense membranes, which had a thickness of 1 ± 0.3 µm as determined by scanning electron microscopy (SEM) measurements. Its cross-linking was achieved by heating the membrane at 110 °C for 120 min. The choice of PAN substrate was due to the fact that the Sulzer Chemtech company uses a polyacrylonitrile-based substrate for membrane production, and the choice of the UPM-20 substrate was due to its good mechanical properties, as determined by earlier studies [23,38].

In Table 1, the names of the PVA-based membranes are presented in abbreviated form.

### 2.3. Methods

#### 2.3.1. IR Spectroscopy

IR spectroscopy was performed using the attenuated total reflection (ATR) methodology in the range of 650–4000 cm^−1^ at 25 °C on a BRUKER-TENSOR 27 spectrometer (Bruker Optik GmbH, Ettlingen, Germany) to determine the nature of the interaction between PVA and PAH.

#### 2.3.2. Scanning Electron Microscopy (SEM)

A Zeiss Merlin SEM (Carl Zeiss SMT, Oberhochen, Germany) was used to obtain SEM micrographs of the membrane cross-sections. The membranes were submerged in liquid nitrogen for 5 min and fractured perpendicular to the surface. The prepared specimens were observed using SEM at 1 kV.

#### 2.3.3. Atomic Force Microscopy (AFM)

The topography of the surfaces of membranes was studied on an NT-MDT NTegra Maximus atomic force microscope (NT-MDT Spectrum Instruments, Moscow, Russia) with standard silicon cantilevers with a rigidity of 15 N∙m^−1^ (“NT-MDT”, Russia) in tapping mode.

#### 2.3.4. X-Ray Diffraction

The characterization of the membrane structure was carried out using a Bruker D8 DISCOVER diffractometer (Bruker, Karlsruhe, Germany) (40 kV, CuKα radiation, step size 0.05°, 40 mA,) at room temperature in the Bragg–Brentano geometry. The obtained small-angle X-ray scattering (SAXS) data were analyzed with the ATSAS software package [39].

#### 2.3.5. Contact-Angle Determination

Contact angles of membrane surface were measured by the sessile drop method as described in Reference [40] to study the stability of PEL bilayers. The measurements were carried out only from the side of the thin selective layer to study the surface characteristics of the membranes.

#### 2.3.6. LbL Deposition Technique

PAH (10^−2^ mol/L) and PSS (10^−2^ mol/L) were used as polyelectrolyte solutions. The pH for the PAH solutions was adjusted to 4 because this polyelectrolyte was fully ionized at this value.

**Figure 1:**
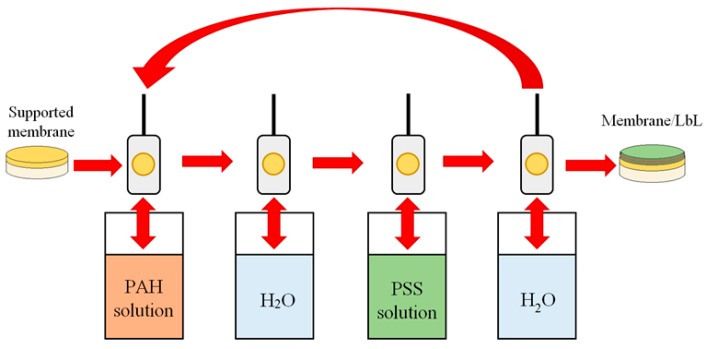
The scheme of LbL deposition technique.

The multilayer deposition of PEL layers was carried out by ND Multi Axis Dip Coaters ND-3D 11/5 (Nadetech, Noáin (Navarra), Spain) with a wide speed immersion range (from 1 mm∙min^−1^ to 2000 mm∙min^−1^). The membrane was clamped on a special support and immersed into solutions for different times. The procedure was as follows (shown in Figure 1): firstly, the membrane was deposited into a polycation solution of PAH for 10 min. Next, it was removed and rinsed thoroughly with water for 1 s 15 times, 5 s three times, and 15 s one time, successively. Then, the membrane was immersed in PSS solution for 10 min, after which the same water rinsing process of the membrane was repeated. In this way, one PEL bilayer was created on the membrane surface. The required number of additional bilayers was deposited according to a similar scheme. When the PAH polycation was previously introduced into the PVA matrix, the PSS polyanion would be firstly deposited on the membrane surface. The optimum number of PEL bilayers applied to the surface of the membrane was 10, because the deposition of fewer or more than 10 bilayers did not lead to the improvement of membrane transport properties [23].

#### 2.3.7. Pervaporation Experiments

Transport properties were studied using a pervaporation laboratory cell in steady-state regime at different temperatures (20, 30, 45, 60 °C) [38]. The composition of permeate and feed was analyzed by gas chromatography using a SHIMADZU GC-2010 chromatograph (SHIMADZU, Nancy, France) equipped with an HP-PLOT/U column (Agilent J&W GC Columns, Nancy, France) and a thermal conductivity detector.

The membrane permeation flux J (kg/(m^2^h)) was determined to be the amount of liquid transported through a unit of the membrane area per hour and was calculated as follows [41]:

$$J=\frac{W}{A\times t}$$

where *W* (kg) is the weight of the liquids that permeated the membrane, *A* (m^2^) is the effective membrane area, and *t* (h) is the measurement time.

The separation factor (β) was calculated as follows [42]:

β=yiyjxixj

where *y_i_* and *y_j_* are the weight fractions of components *i* and *j* in the permeate, and *x_i_* and *x_j_* are the weight fractions of components *i* and *j* in the feed.

Each measurement was performed at least three times to ensure good accuracy of the transport parameters, and the average value was recorded for later analysis. The mean accuracies for the transport parameters were as follows: for a dense membrane, ±0.5% for water content in the permeate and ±5% for permeation flux; for supported membranes, ±1% for water content in the permeate and ±6% for permeation flux.

#### 2.3.8. The Standard Porosimetry Method

The porosimetry of the commercial porous substrates PAN and UPM-20 was studied by the standard porosimetry method on a Porosimeter 3.1 instrument at 30 ± 0.5 °C. The samples of the membranes were prepared in the form of a “tablet” of regular shape with a diameter of up to 23 mm. *n*-Octane was used as the reference liquid.

#### 2.3.9. Filtration Performance of Porous Substrates

**Figure 2:**
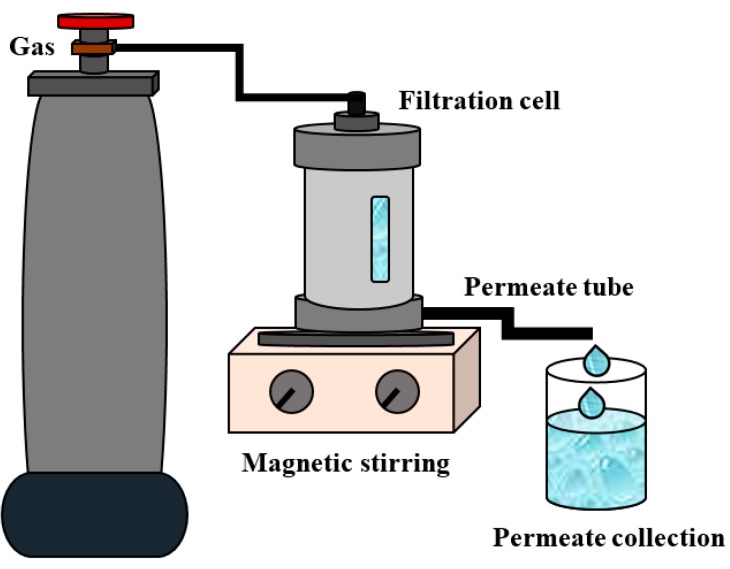
The scheme of the filtration set-up.

Water flux measurements and rejection calculations for commercial modified porous substrates (PAN and UPM-20) were carried out in a custom-made stirred dead-end filtration system (Figure 2) at 25 °C and at the average transmembrane pressure of 2 bar. Bovine serum albumin (BSA) was used as a protein foulant and prepared as 0.5 g/L in buffered solution (pH = 7).

The permeation flux (J) of water was calculated by the following equation [43]:

$$J=\frac{V}{A\times \Delta t}$$

where V (L) is the permeate volume, A (m^2^) is the effective membrane area, and Δt (h) is the permeation time.

The BSA content in the permeate was evaluated by spectrophotometry on a Spectrophotometer SF-102 (NPO INTERPHOTOPHYSICS, Moscow, Russia) (at a wavelength of 280 nm corresponding to the maximum value for the protein).

The BSA rejection (R, %) was calculated by the following equation:

$$R=\left(1-\frac{c_{p}}{c_{f}}\right)\times 100\%$$

where *c_p_* and *c_f_* are the BSA contents in the permeate and feed (g/L), respectively.

## 3. Results

### 3.1. PVA–PAH Structure Characteristics 

In our previous study [23], it was shown that the PVA-based supported membranes modified by PAH possessed increased permeability, but this effect was not fully rationalized. To investigate the influence of the PAH introduction on PVA membrane properties, a set of dense membranes were prepared and studied by IR spectroscopy and scanning electron microscopy for the first time.

**Figure 3:**
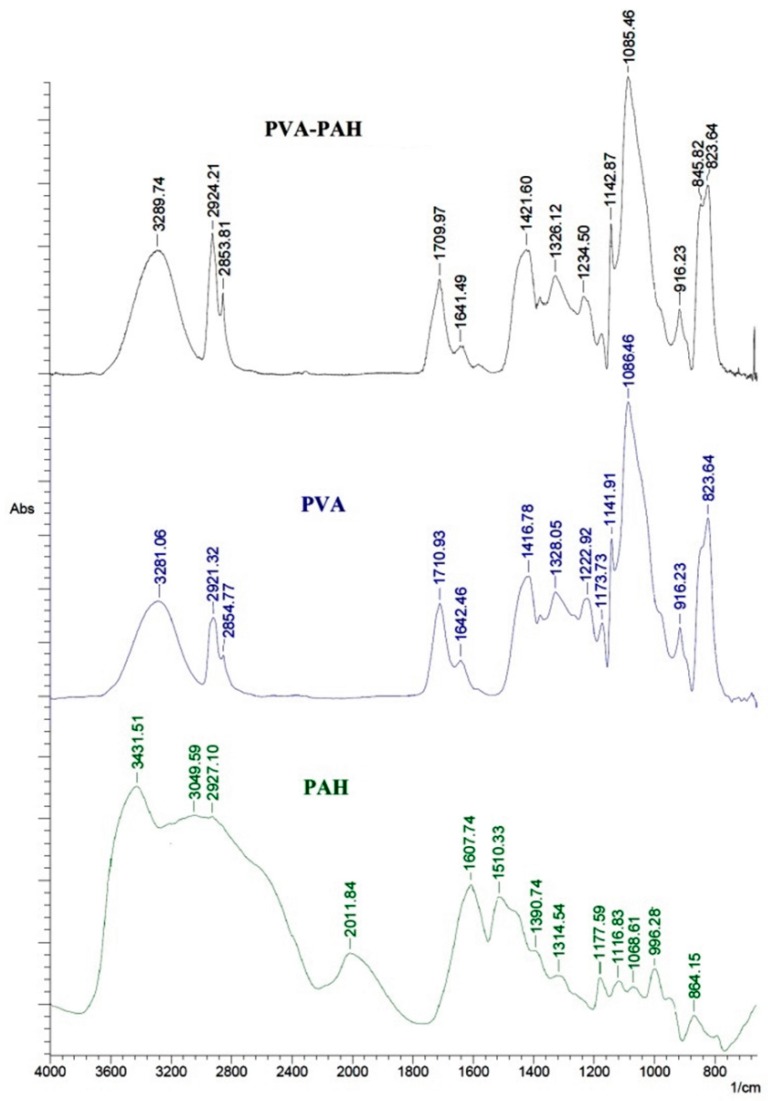
The IR spectra of PAH (powder), chemically cross-linked PVA and PVA–PAH dense membranes.

Figure 3 presents the IR spectra of pristine PAH (powder), and chemically cross-linked membranes based on PVA and a PVA–PAH (4.7%) polymer blend.

The spectrum for the modified PVA–PAH membrane was significantly different from the spectrum of PAH due to its small addition. Comparing the spectra of PVA and PVA–PAH membranes, the following changes were observed: two peaks at 2924 and 2854 cm^−1^ in the spectrum of the PVA–PAH membrane had a higher intensity compared to the peaks of the PVA membrane (at 2921 and 2855 cm^−1^) (Figure 3). This pair of peaks corresponded to asymmetrical and symmetrical valence vibrations of CH_2_, respectively. During the modification of PVA by PAH, the peaks at 1417 cm^−1^ (corresponding to asymmetric deformation vibrations of the CH group) and at 1223 cm^−1^ (corresponding to the region of deformation vibrations of CH groups) of the PVA membrane shifted to 1422 and to 1235 cm^−1^ for the PVA–PAH membrane. In interpreting this phenomenon, it can be concluded that the characteristics of the CH bond changed and, perhaps, there are intermolecular interactions related to the hydrophobic parts of the PVA molecule after modification by the polyelectrolyte PAH [44]. 

It should be noted that, for the chemically cross-linked PVA membrane, i.e., with the addition of maleic acid (MA) and subsequent heating at 110 °C for 120 min, two structures could be formed according to the previous studies [37,45]. According to the obtained spectra, it is possible to form various bonds between PVA and MA, PVA and PAH, and PAH and MA (ether and ester bonds, hydrogen bonds, etc.). Thus, the establishment of a structure using spectral methods of analysis is significantly complicated.

**Figure 4:**
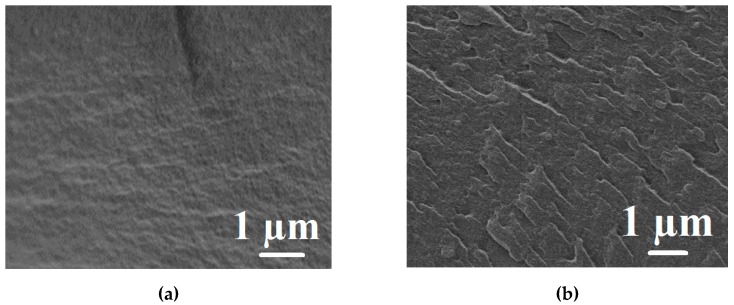
The cross-sectional SEM micrographs of chemically cross-linked dense PVA (**a**) and PVA–PAH (**b**) membranes.

SEM was applied to visualize the effect of PAH introduction into the PVA matrix on the inner morphology of membranes. The corresponding cross-sectional micrographs of PVA and PVA–PAH membranes are presented in Figure 4.

It was shown by the cross-sectional SEM micrographs (Figure 4) that the degree of roughness of the dense PVA–PAH membrane increased significantly after the introduction of PAH into the PVA matrix (Figure 4b). The PVA membrane has a relatively smooth and uniform cross-section structure, while the introduction of PAH into the PVA matrix leads to the appearance of plastic deformations on the cross-section of the modified membrane, which can provide significant changes in transport properties compared to a pristine PVA membrane.

Additionally, the structure of the obtained PVA and PVA–PAH membranes was characterized by SAXS, and the changes in the surface properties for PVA-based membranes due to the modification by PAH were evaluated by the static sessile drop method to measure the contact angles of water. The calculated results from SAXS data and the values of the obtained contact angles for the dense membranes are presented in Table 2.

From the data of Table 2, it was determined that the radius of gyration and Porod volume slightly increased when PAH was added to the initial polymer, reflecting a higher degree of cross-linking. The introduction of PAH to the PVA matrix led to the decrease in contact angle from 67 to 61°, which indicated the hydrophilization of PVA–PAH membrane surface due to the rise in quantity of polar groups on the surface of the membrane. These changes in the structure and surface of the PVA membrane during the modification by PAH could affect the transport properties of the obtained membranes.

### 3.2. Transport Properties of Modified PVA Membranes

The study of chemically cross-linked dense membranes allows an accurate description of the transport characteristics of the selected PVA polymer and PVA–PAH polymer blend, since the effect of substrate and defects in the selective layer can be excluded. To study the influence of the nature of the applied substrate for the preparation of supported mixed matrix PVA membranes, a new commercial porous substrate based on polyacrylonitrile (PAN) was used for the deposition of a thin selective layer based on PVA or PVA–PAH polymer blend. Further surface modification by LbL assembly (the deposition of the PSS/PAH bilayers) for pervaporation dehydration of i-PrOH (20 wt % water) was carried out to compare with the supported membranes on the porous substrate from aromatic polysulfone amide (UPM-20) studied earlier [23].

#### 3.2.1. Transport Properties of PVA and PVA–PAH Membranes

**Figure 5:**
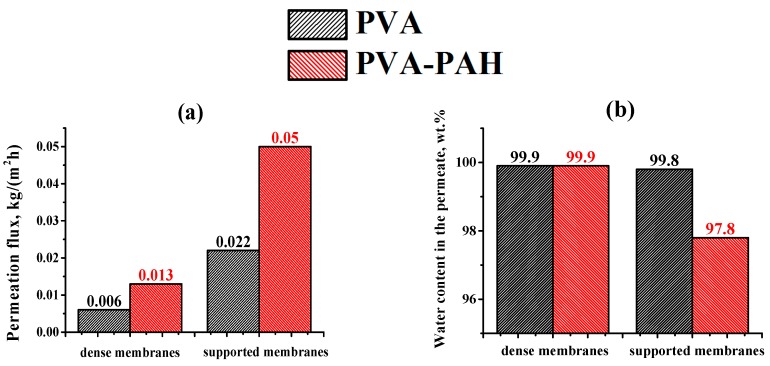
Dependence of permeation flux (**a**) and water content in the permeate (**b**) on types (dense or PAN-supported) of membranes based on PVA and PVA–PAH (4.7%) during pervaporation of an i-PrOH (80 wt %)/water (20 wt %) mixture at 20 °C.

Two types of membranes were developed and investigated: dense membranes based on PVA and PVA–PAH polymer blend, and supported membranes with the same compositions of the thin selective layer on a commercial porous PAN substrate. Their transport properties were studied in the separation of an industrially significant isopropanol (i-PrOH) (80 wt %)–water (20 wt %) mixture by pervaporation at 20 °C. The comparison of transport properties of obtained membranes during the isopropanol dehydration is presented in Figure 5.

The pervaporation results of Figure 5a demonstrate that the introduction of PAH into the PVA matrix led to a ~2.2-fold increase in permeation flux at high selectivity with respect to water (Figure 5b) (water content in the permeate not less than 97.8 wt %) for both membrane types (dense and supported) compared to the corresponding type of pristine PVA membrane. This result was likely due to changes in inner membrane structure demonstrated by SEM (Figure 4) and the increase in hydrophilicity of the PAH-containing membrane surface (Table 2). It should be noted that the creation of the supported membrane based on the PVA–PAH (4.7%) polymer blend with PAN substrate (PVA–PAH^PAN^) led to a significant increase in the permeation flux (~4-fold) compared to the dense PVA–PAH membrane with a slight decrease in water selectivity (a reduction of water content in the permeate from 99.9 to 97.8 wt %) (Figure 5a,b). The increase in permeability and decrease in selectivity compared to unmodified PVA membranes was also noted for the PVA–PAH supported membrane when the substrate based on aromatic polysulfone amide (UPM-20) was used [23] (Table 3).

It was found that the application of PAN substrate for PVA-based supported membranes led to a higher water content in the permeate and lower permeation flux compared to the application of UPM-20 substrate during pervaporation of an i-PrOH (80 wt %)/water (20 wt %) mixture at 20 °C (Table 3). The investigation of this effect is presented in Section 3.3.

**Figure 6:**
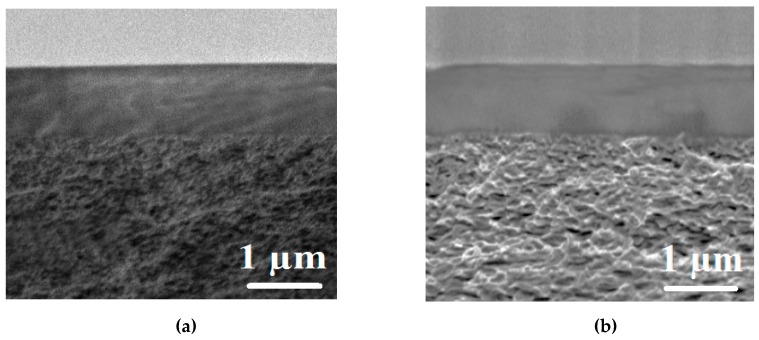
The SEM micrographs of cross-section for (**a**) PVA–PAH^PAN^ and (**b**) PVA–PAH^UPM^ membranes.

To study the inner structure of supported membranes and to estimate the thickness of a selective layer, scanning electron microscopy (SEM) was used. Figure 6 demonstrates the cross-sectional micrographs of the supported membranes based on the PVA–PAH polymer blend on PAN and UPM-20 substrates. 

A uniform nonporous structure of the dense selective PVA–PAH top layer with good adhesion to spongy porous PAN and UPM-20 substrates is presented on the micrographs (Figure 6). Figure 6 demonstrates that the thickness of the top selective layer is determined as ∼1 ± 0.3 μm. Notably, a similar SEM images were obtained for the pristine PVA membranes (not shown here).

**Figure 7:**
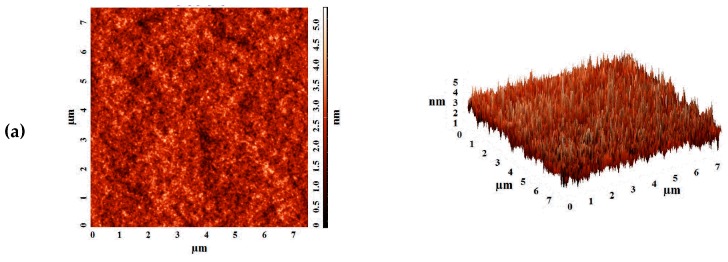

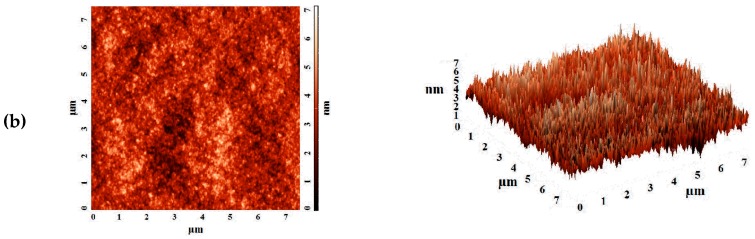
The AFM images obtained in tapping mode for (**a**) PVA–PAH^PAN^ and (**b**) PVA–PAH^UPM^ membranes.

To study the surface topography of supported membranes, atomic force microscopy (AFM) was used. Figure 7 demonstrates AFM images of the skin layer surface based on polymer blend PVA–PAH of the supported membranes on PAN and UPM-20 substrates with a scan size of 7.5 × 7.5 μm. 

Based on AFM images, the roughness characteristics in terms of root-mean-squared surface roughness (R_q_) and average roughness (R_a_) for supported PVA–PAH^PAN^ and (b) PVA–PAH^UPM^ membranes were calculated (Table 4). These roughness characteristics can strongly affect the sorption of the feed components on the membrane surface and the membrane permeability.

It was shown that the deposition of the selective layer based on PVA–PAH on the porous UPM substrate led to a more pronounced surface roughness of supported membrane compared to the deposition on PAN substrate (Table 4). The average surface roughness (R_a_) for PVA–PAH^UPM^ membrane increased 1.4-fold compared to the average surface roughness of PVA–PAH^PAN^ membrane (0.48 nm). The increase in surface roughness, providing a large surface area for contact with the feed, is one of the factors that leads to facilitated sorption of penetrants and promotes faster penetration of the feed components. This results in significantly improved membrane permeability, which is in agreement with the pervaporation data (Table 3).

Due to the obtained high water content in the permeate for the PVA–PAH^PAN^ membrane, it was chosen for further surface modification by LbL deposition of polyelectrolyte PSS/PAH bilayers with the aim of further improvement of transport parameters and investigation of the influence of the use of PAN substrate on stability and transport properties of the developed membrane.

#### 3.2.2. Transport Properties of Membranes Modified by LbL Assembly

One of the perspective methods to functionalize the membrane surface in order to improve the transport membrane parameters is the deposition of polyelectrolytes via the layer-by-layer (LbL) technique. The application of this method is very relevant and allows the use of different amounts of nanoscale layers of oppositely electrically charged polyelectrolytes (polycation and polyanion) to control the modification of the surface (change in hydrophilicity, creation of charged surface, etc.). The surface of the supported PVA-based membranes was coated by 10 and 20 bilayers of polyelectrolytes: poly(allylamine hydrochloride) as polycation and poly(sodium 4-styrenesulfonate) as polyanion. The bulk modification of PVA membranes by the introduction of PAH was carried out for better adhesion of PEL bilayers with the membrane surface. The transport properties of membranes modified by PEL were studied in pervaporation separation of a mixture of i-PrOH (80 wt %) and water (20 wt %) at 20 °C (Table 5).

The presented data in Table 5 demonstrate that, for all PAN-supported membranes modified by LbL deposition, there is 99.9 wt % water in the permeate, which indicates very high and improved selective properties for the developed membranes compared to the pristine supported membranes (Figure 5b). Moreover, the coating of 10 PEL bilayers on the surface of supported membranes based on PVA and PVA–PAH (4.7%) increased the permeation flux ~1.5-fold and ~1.2-fold, respectively, compared to the supported membranes without LbL modification (Figure 5a, Table 3), because of the formation of small hydrophilic mashes, which induced penetration of the more polar component, i.e., water [36].

For further improvement of the transport properties of the supported PVA–PAH^PAN^ membrane, it was decided to increase the number of polyelectrolyte bilayers to 20 and to identify the dependence of transport characteristics on the bilayer amount for the dehydration of i-PrOH by pervaporation, because the deposition of fewer than 10 bilayers did not lead to the improvement of transport properties of membranes. The data in Table 5 demonstrate that an increase of polyelectrolyte bilayers up to 20 for the PVA–PAH/LbL-20^PAN^ membrane led to the reduction in permeation flux (~1.4-fold) at the same water content in the permeate compared to the membranes with 10 PEL bilayers (PVA–PAH/LbL-10^PAN^). This effect was due to an increase in the thickness of the polyelectrolyte layer on the membrane surface, which limited the diffusion of the separated components to the selective layer based on PVA, ultimately leading to a decrease in the membrane flux [23]. Thus, 10 PEL bilayer coatings on the PVA–PAH membrane surface were chosen as the optimal condition of surface modification by LbL deposition. It should be noted that this developed PVA–PAH/LbL-10^PAN^ membrane possessed significantly higher selectivity properties (99.9 wt % water content in the permeate) compared to the PVA–PAH/LbL-10^UPM^ supported membrane on the UPM-20 substrate (Table 5). 

Thus, the data of Table 3 for PVA–PAH membranes without LbL surface modification demonstrated that the permeation flux for the PAN-supported membrane (PVA–PAH^PAN^) decreased ~4.5-fold compared with the permeation flux of the PVA–PAH^UPM^ membrane on the UPM-20, while the water content in the permeate for the PVA–PAH^PAN^ membrane deposited on the PAN was much higher compared to the UPM-supported membrane. The same trend persisted after the deposition of 10 PEL bilayers on the surface of the membranes. However, it should be emphasized that the surface modification by 10 PEL bilayers led to a significant decrease in water content in the permeate (68.4 wt %) with a slight increase in permeation flux (0.261 kg/(m^2^h)) of the supported membrane on UPM-20 (PVA–PAH/LbL-10^UPM^) compared to the PVA–PAH membrane without surface modification on UPM-20 (PVA–PAH^UPM^) (92.8 wt % water in the permeate, 0.224 kg/(m^2^h)). On the other hand, for the PVA–PAH supported membrane on PAN (PVA–PAH/LbL-10^PAN^), both membrane parameters were improved (99.9 wt % water in the permeate, 0.061 kg/(m^2^h)) as compared to the supported PVA–PAH^PAN^ membrane without surface modification (97.8 wt % water in the permeate, 0.050 kg/(m^2^h)). Thus, the obtained data demonstrate that the transport characteristics of the supported membranes depend significantly on the choice of the commercial porous substrate.

### 3.3. Influence of the Substrate on the Mass Transport

Despite the generally accepted opinion that the porous substrate of supported membranes does not significantly affect the mass transfer of low-molecular-weight components in pervaporation, the substrate polymer type, as well as substrate porosity, can have a significant effect on the transport properties of the supported membrane [46,47,48]. In this section, to understand the mechanism of mass transfer in pervaporation, the characteristics of commercial porous substrates PAN and UPM-20 were studied by SEM, AFM, the standard porosimetry method, contact-angle measurements, and ultrafiltration experiments. 

**Figure 8:**
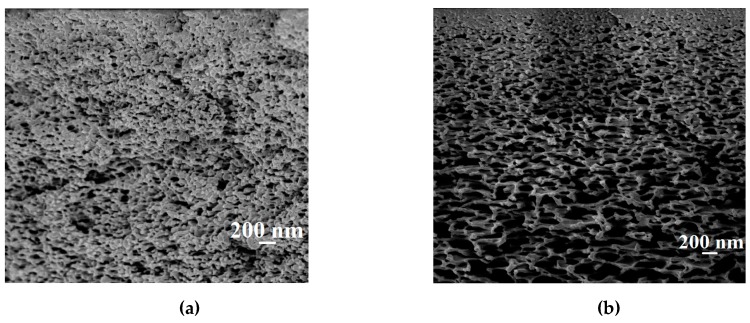
The SEM cross-sectional micrographs for the commercial porous (**a**) PAN and (**b**) UPM-20 substrates.

The inner structure of commercial membranes was studied by SEM for the evaluation of inner morphology. The SEM cross-sectional micrographs of industrial PAN and UPM-20 substrates are presented in Figure 8. 

The presented microphotographs in Figure 8 demonstrate that the bulk porosity of the PAN substrate is much smaller than for the UPM-20 substrate, which is one of key factors for permeation flux decrease and the increase in water content in the permeate with the use of a PAN membrane as the substrate compared to the UPM-20 substrate [46]. 

To study porosity of commercial PAN and UPM-20 substrates, the standard porosimetry method was applied. The obtained data of characteristics of size of pore distribution are presented in Table 6.

As it can be seen from the data presented in Table 6, the porosity parameters for the UPM-20 substrateare higher than for the PAN substrate (in agreement with SEM data (Figure 8)).

The study of the rejection of bovine serum albumin by ultrafiltration was carried out in order to study the surface porous layer of commercial substrates. The ultrafiltration results are presented in Table 7.

Via the ultrafiltration experiment, it was demonstrated that the permeation flux of water for the UPM-20 substrate significantly exceeded the water permeation flux for the PAN (Table 7) in agreement with the porosity, SEM, and pervaporation data. However, for both commercial membranes, the values of rejection (R) after the ultrafiltration of BSA were equal (~99%), which indicated that the pore size on the skin surface layer of the substrates (pores responsible for retaining the solute) was similar (in agreement with membrane passports) and they retained BSA equally well. However, it should be noted that the bulk porosity of these substrates, as measured by the standard porosimetry method, was significantly different (Table 6), which to a greater extent affected the difference in permeation fluxes of supported membranes during pervaporation. 

**Figure 9:**
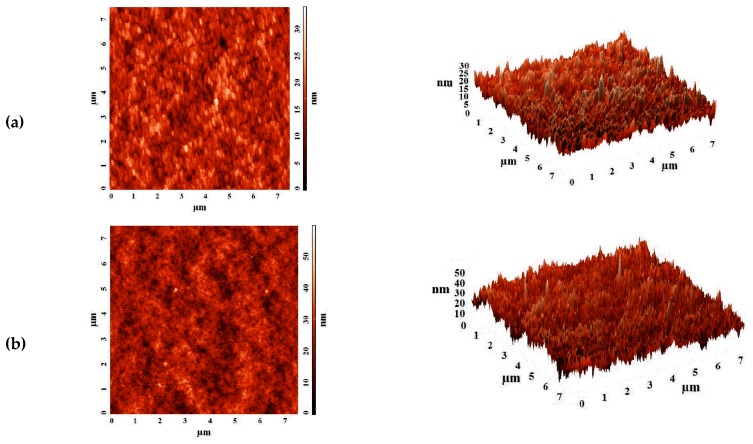
The AFM images obtained in the tapping mode for commercial porous (**a**) PAN and (**b**) UPM-20 substrates.

Atomic force microscopy (AFM) and the sessile drop method for measuring the contact angle of water were used to evaluate the difference in surface morphology of the commercial membranes. The AFM images of the skin layer surface of the PAN and UPM-20 substrates with a scan size of 7.5 × 7.5 μm are presented in Figure 9. 

The roughness characteristics calculated based on AFM images, as well as contact angles of water measured by the sessile drop method for assessing the hydrophilic–hydrophobic surface properties, for PAN and UPM-20 substrates are presented in Table 8. 

From the AFM images (Figure 9) and roughness characteristics (Table 8), it was demonstrated that the commercial UPM-20 membrane (the average surface roughness (R_a_) was equal to 4.48) had a rougher surface compared to the PAN substrate, which could affect the roughness of a thin selective layer based on the PVA–PAH polymer blend deposited on it (Table 4). Also, the UPM-20 substrate was more hydrophobic (contact angle of water 63°) compared to the PAN (48°). The lower contact angle of the PAN substrate caused better affinity of this substrate to water and, in combination with the low porosity of substrate (SEM, ultrafiltration, and porosity data), resulted in better selectivity with respect to water of PAN-supported membranes, while the more hydrophobic UPM substrate, which possessed higher porosity, was more favorable for the penetration of both separated components (isopropanol and water). Additionally, different substrate polymer types may influence the crystallinity of the selective dense layer based on PVA, which was reflected in the transport properties of the pervaporation membrane.

Thus, based on the obtained pervaporation data on the separation of the isopropanol–water mixture and data on the characterization of commercial porous substrates, it can be concluded that a supported membrane with a thin selective layer based on PVA–PAH deposited on porous PAN substrate and modified with 10 PEL bilayers via LbL deposition (PVA–PAH/LbL-10^PAN^ membrane) has the best selective properties with respect to water. On the other hand, the UPM-20 substrate, characterized by a much higher porosity, did not limit the mass transport of the low-molecular-weight component, which logically favored a higher permeance [46]. Nevertheless, at the same time, the water selectivity was reduced due to the coupling effect of several parameters: a more open pore structure, a rougher and more hydrophobic surface, and facilitated diffusion of iPrOH in UPM-20 (compared to PAN).

### 3.4. Investigation of PAN-Supported PVA and PVA–PAH Membranes at Different Temperatures

**Figure 10:**
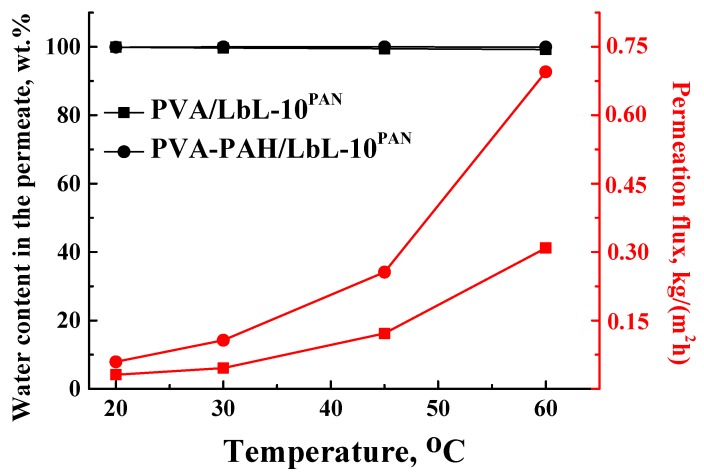
Dependence of the water content in the permeate and the permeation flux on temperature (20, 30, 45, 60 °C) for the pervaporation of an isopropanol (80 wt %)–water (20 wt %) mixture by supported PVA/LbL-10^PAN^ and PVA–PAH/LbL-10^PAN^ membranes.

For investigation of the industrial application of a supported PVA-based membrane modified by 4.7 wt % PAH and 10 bilayers of PEL (PVA–PAH/LbL-10^PAN^) with the best transport properties for pervaporation isopropanol dehydration, it was decided to estimate the stability of this membrane for the separation of an isopropanol–water (20 wt % water) mixture at different temperatures (20, 30, 45, 60 °C), since most industrial processes were carried out at elevated temperatures. The transport properties of the PVA/LbL-10^PAN^ and PVA–PAH/LbL-10^PAN^ membranes are presented in Figure 10.

The data of Figure 10 demonstrate that the permeation flux of the supported membranes increases with the rise in temperature, while the water content in the permeate remaines constant even at elevated temperature (≥99.22 wt %). The permeation flux of the modified PVA–PAH/LbL-10^PAN^ membrane was much higher (~2-fold) compared to the permeation flux of the PVA/LbL-10^PAN^ membrane due to the bulk modification by PAH. It should be noted that the water content in the permeate for both membranes almost did not change during the experiment at elevated temperatures (99.99–99.22 wt % water in the permeate), indicating the stability and high selectivity of the developed membrane with respect to water.

It was shown that the selective properties of the supported PVA–PAH/LbL-10 ^PAN^ membrane remained unchanged up to 60 °C (Figure 10) for the separation of the isopropanol–water mixture (20 wt % water). 

### 3.5. Comparison of PVA–PAH/LbL-10^PAN^ Membrane with PVA-Based Membranes 

The performance of the PVA–PAH/LbL-10^PAN^ membrane was compared with mixed matrix PVA membranes described in the literature for isopropanol pervaporation dehydration and with a commercial analogue membrane “PERVAP^TM^ 1201” (SULZER) that contained PAN as a substrate and was designed for the dehydration of water–organic mixtures (up to 80 wt % water in the feed). Comparison of the transport properties of PVA-based membranes for pervaporation of isopropanol/water system was done in terms of the separation factor and the permeation flux under conditions close to the present study and presented in Table 9.

The data of Table 9 demonstrate that the developed PVA–PAH/LbL-10^PAN^ membrane exhibites a very high separation factor (39,969) and high permeation flux (0.713 kg/(m^2^h)) for the pervaporation of an isopropanol–water (80/20 wt %) mixture at 60 °C. These values were much greater than obtained for PVA membranes in previous studies. Only the “PERVAP^TM^ 2510” membrane and the PVA membrane modified by graphene oxide quantum dots possessed higher permeation flux (0.857 and 1.502 kg/(m^2^h), respectively) but with significantly decreased separation factors (1128 and 117, respectively) for pervaporation of an isopropanol/water mixture compared to the PVA–PAH/LbL-10^PAN^ membrane. In addition, the permeation flux of the developed PVA–PAH/LbL-10^PAN^ membrane in this study was ~4.5-fold higher than the permeation flux of the commercial analogue “PERVAP^TM^ 1201” with the same separation factor value (39,969) for pervaporation dehydration of isopropanol (20 wt % water) at 60 °C. These results indicate the prospective industrial applications of this membrane for the dehydration of water–organic mixtures.

### 3.6. Study of the Stability of Top Nanolayers Deposited via LbL Technique on the Surface of PVA–PAH/LbL-10^PAN^ Membrane

The contact angles measured by static sessile drop method and scanning electron microscopy (SEM) were used to study and verify the stability of the polyelectrolyte multilayer on the surface of the supported PVA–PAH/LbL-10^PAN^ membrane. 

By measuring the contact angles, it was shown that the values of the water contact angle for the membrane surface remained unchanged and were equaled 74° ± 3° before and after pervaporation of isopropanol (80 wt %)–water (20 wt %) at 20 °C, which indicated that the properties of the membrane surface did not change after contact with the separated mixture and proved the stability of the polyelectrolyte layers on the surface of supported membrane during the process of dehydration by pervaporation.

**Figure 11:**
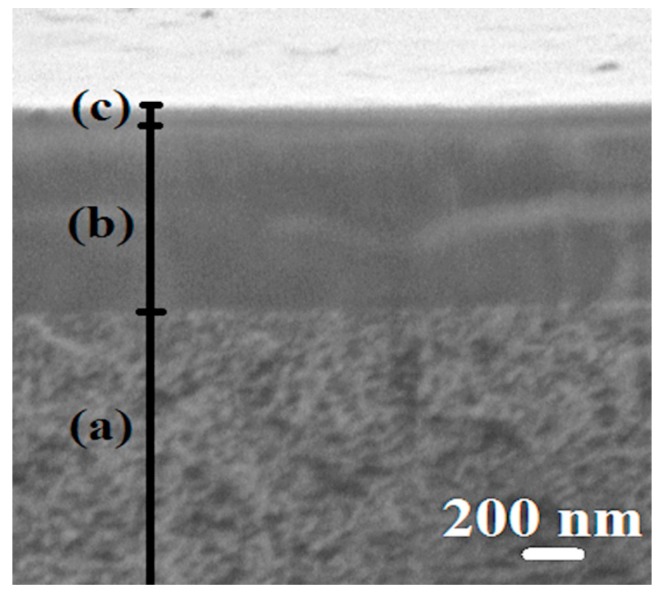
The cross-sectional SEM micrograph of supported PVA–PAH/LbL-10^PAN^ membrane after pervaporation: (**a**) the region of the porous PAN substrate, (**b**) the selective thin nonporous PVA–PAH layer, and (**c**) the layer of polyelectrolytes (PSS, PAH).

Cross-sectional SEM micrographs of the PVA–PAH/LbL-10^PAN^ membrane were taken before and after pervaporation for the confirmation of the stability of 10 PEL bilayers. These micrographs were similar. In this connection SEM micrograph of cross-section of the PVA–PAH/LbL-10^PAN^ membrane only after pervaporation is presented in Figure 11.

On the cross-sectional SEM micrograph of the PVA–PAH/LbL-10^PAN^ membrane (Figure 11), three areas can be observed: (a) the region of the porous PAN substrate, (b) the selective thin nonporous PVA-PAH layer, and (c) the layer of polyelectrolytes (PSS, PAH). The size of the polyelectrolyte multilayer was determined as ~50 nm by SEM. It was found that PEL layer on the membrane surface was stable and not washed away after the experiment on the separation of the i-PrOH/water mixture.

Thus, these investigations confirm that a novel green PAN-supported membrane based on a PVA–PAH (4.7%) polymer blend and modified by 10 PEL layers (PVA–PAH/LbL-10^PAN^) have improved transport characteristics compared to a membrane based on pure PVA, as well as highly selective properties with respect to water that are preserved and unchanged at elevated temperatures. Also, the stability of the composition and the invariability of the properties of the developed supported membrane after pervaporation were confirmed, which indicated the promise of using this membrane in industrial processes for the dehydration of organic substances.

## 4. Conclusions

In this work, the effect of the introduction of the widely used cationic polyelectrolyte poly(allylamine hydrochloride) (PAH) into a PVA matrix for understanding the mechanism of mass transfer in pervaporation dehydration was studied. It was shown that the modification of PVA by PAH led to a significant change in the physical membrane properties; it changed the internal and surface morphology of membranes, increased the hydrophilicity of the surface, and led to a change in the structural characteristics of the membranes. These phenomena significantly affected the transport properties of the developed PVA-based membranes and caused a ~2-fold increase in permeation flux while preserving the high water content in the permeate (99.9 wt %) for the dense PVA–PAH membrane compared to the pristine dense PVA membrane during the separation of an isopropanol–water (80/20 wt %) mixture by pervaporation at 20 °C.

Additionally, the influence of the nature of the used commercial porous substrates (PAN and UPM-20) for the preparation of supported PVA-based membranes with bulk (the introduction of PAH into the PVA matrix) and surface (the deposition of the PSS/PAH bilayers) modifications for pervaporation dehydration of i-PrOH (20 wt % water) was studied.

It was found that the application of PAN substrate for PVA-based membranes with or without 10 PEL bilayers led to much higher water content in the permeate and lower permeation flux (~4-fold) compared to the application of the UPM-20 substrate during pervaporation of an i-PrOH (80 wt %)/water (20 wt %) mixture at 20 °C. It should be underlined that the effect of surface modification via LbL deposition of 10 bilayers for supported PVA–PAH membranes was quite different depending on the used substrate. Specifically, the coating of 10 bilayers (PSS/PAH) on the surface of the PVA–PAH^UPM^ membrane led to a significant decrease in water content in the permeate with a slight increase of permeation flux compared to the PVA–PAH membrane without surface modification on UPM-20 (PVA–PAH^UPM^). For the PAN-supported membrane, there was another trend; both membrane transport parameters were improved after the surface modification with 10 bilayers (PSS/PAH). Thus, it was demonstrated that the transport characteristics of the supported membranes depended significantly on the choice of the commercial porous substrate (UPM-20 and PAN) because of differences in the chemical nature of the substrate material, pore size, and porosity.

Based on the obtained results, it could be concluded that a supported membrane with a thin selective layer based on a polymer blend of PVA–PAH deposited on a porous PAN substrate and modified with 10 PEL bilayers via LbL deposition (PVA–PAH/LbL-10^PAN^ membrane) exhibited highly selective properties with respect to water (≥99 wt % water in the permeate) at different temperatures (20, 30, 45, 60 °C). Moreover, this membrane also had a permeation flux ~4.5-fold higher than that of the commercial analog PERVAP^TM^ 1201 (Sulzer) with the same selectivity level for the pervaporation dehydration of i-PrOH (20 wt % water) at 60 °C. This developed membrane is promising for industrial application in the dehydration of organic solvents.

## Figures and Tables

**Table 1:** The polyvinyl alcohol (PVA) membrane samples.

Membranes	Type	Thicknes µm	Weight Content, %		Surface Modification	
			Cross-Linker (Maleic Acid (MA))	Bulk Modifier poly(Allylamine Hydrochloride) (PAH)	Quantity of Bilayers	Type of Polyelectrolytes (PEL)
PVA	Dense	45	35	-	-	-
PVA–PAH	Dense	45	35	4.7	-	-
PVA^PAN*^	Supported	1	35	-	-	-
PVA–PAH^PAN^	Supported	1	35	4.7	-	-
PVA/LbL-10^PAN^	Supported	1	35	-	10	PSS, PAH
PVA–PAH/LbL-10^PAN^	Supported	1	35	4.7	10	PSS, PAH
PVA–PAH/LbL-20^PAN^	Supported	1	35	4.7	20	PSS, PAH
PVA^UPM^	Supported	1	35	-	-	-
PVA–PAH^UPM^	Supported	1	35	4.7	-	-
PVA/LbL-10^UPM^	Supported	1	35	-	10	PSS, PAH
PVA–PAH/LbL-10^UPM^	Supported	1	35	4.7	10	PSS, PAH

* To simplify the designation of membranes, the types of the applied commercial substrates for the preparation of supported membranes are indicated in the form of an upper index.

**Table 2:** The calculated results from SAXS and contact angles for dense PVA and PVA–PAH membranes.

Membrane	R_G_, nm	Porod Volume, 10^5^ nm^3^	Contact Angle of Water, °
PVA	46.7	9.76	67
PVA–PAH	47.2	9.93	61

**Table 3:** Pervaporation of an isopropanol (80 wt %)/water (20 wt %) mixture at 20 °C by supported PVA–PAH membranes.

Membrane	Permeation Flux, kg/(m^2^h)	Water Content in Permeate, wt %	References
PVA-PAH^PAN^	0.050	97.8	This study
PVA-PAH^UPM^	0.224	92.8	[23]

**Table 4:** Surface parameters of the supported PVA–PAH^PAN^ and (b) PVA–PAH^UPM^ membranes.

Membranes	R_q_, nm	R_a_, nm
PVA–PAH^PAN^	0.61	0.48
PVA–PAH^UPM^	0.86	0.68

**Table 5:** Pervaporation of i-PrOH (80 wt %)/water (20 wt %) mixture at 20 °C using supported membranes after LbL modification.

Selective Layer	Substrate	The Number of Polyelectrolyte Bilayers	Permeation Flux, kg/(m^2^h)	Water Content in the Permeate (wt %)	References
PVA	PAN	10	0.032	99.9	This study
**PVA–PAH**	**PAN**	**10**	**0.061**	**99.9**	
PVA–PAH	PAN	20	0.043	99.9	
PVA–PAH	UPM	10	0.261	68.4	[23]

**Table 6:** Porosity characteristics of industrial PAN and UPM-20 substrates.

Porosity Characteristics	Commercial Membrane	
	*PAN*	*UPM-20*
Porosity over weight, cm^3^/g	1.10	2.60
Porosity over volume, cm^3^/cm^3^	0.59	0.78
Meso- and macro-pore surface over weight, m^2^/g	4.74	309.96
Meso- and macro-pore surface over volume, m^2^/cm^3^	2.51	93.40

**Table 7:** The filtration properties of commercial PAN and UPM-20 substrates.

Commercial Substrate	Pressure, Bar	Permeation Flux, L/(m^2^h)	R, %
PAN	2	68	99.07
UPM-20	2	120	99.04

**Table 8:** Surface parameters of the commercial porous PAN and UPM-20 substrates.

Membranes	R_q_, nm	R_a_, nm	Contact Angle of Water, °
PAN	3.36	2.66	48
UPM-20	5.64	4.48	63

**Table 9:** Comparison of transport properties of PVA–PAH/LbL-10^PAN^ membrane with PVA-based membranes for isopropanol dehydration by pervaporation.

Membranes	Water in the Feed (wt %)	Temperature, °C	Separation Factor (β)	Permeation Flux, kg/(m^2^h)	References
PVA–PAH/LbL-10^PAN^	20	60	39,969	0.713	This study
PERVAP^TM^ 1201	20	60	39,969	0.159	
PERVAP^TM^ 2201	15	60	~400	~0.22	[9]
PERVAP^TM^ 2510 (PVA/PAN)	15	60	1128	0.857	[49]
PVA/zeolite (KA, NaA, CaA, NaX)	20	50	233–410	0.179–0.216	[3]
PVA/ZSM-5 (6%)	10	50	66	0.01	[50]
PVA/ γ-aminopropyl-triethoxysilane (APTEOS) (5%)	30	50	407	0.678	[51]
PVA/sodium montmorillonite (Na^+^MMT) clay (10%)	10	60	217	0.184	[52]
PVA/Ag-NaZ (5%)	20	60	~1000	~0.15	[53]
PVA/ZIF-8-NH_2_ (7.5%)	15	40	1200	0.112	[54]
PVA/graphene oxide quantum dots (GOQDs)	30	70	117	1.502	[55]
PVA/ tetraethoxysilane (TEOS) (1%)	30	50	-	~0.16	[56]
PVA/1-*n*-butyl-3-methylimidazolium chloride (BMIMCl) (~9%)	10	50	7389	0.022	[57]
